# Design, Synthesis and Structure-Activity Relationships of Novel Chalcone-1,2,3-triazole-azole Derivates as Antiproliferative Agents

**DOI:** 10.3390/molecules21050653

**Published:** 2016-05-19

**Authors:** Sai-Yang Zhang, Dong-Jun Fu, Xiao-Xin Yue, Ying-Chao Liu, Jian Song, Hui-Hui Sun, Hong-Min Liu, Yan-Bing Zhang

**Affiliations:** 1 Collaborative Innovation Center of New Drug Research and Safety Evaluation, School of Pharmaceutical Sciences, Zhengzhou University, Zhengzhou 450001, China; celeron16@163.com (S.-Y.Z.); Fudongjun@gs.zzu.edu.cn (D.-J.F.); zzulyc@163.com (Y.-C.L.); mumuandzz@163.com (J.S.); 15937152262@163.com (H.-H.S.); 2 Henan Medical College, Zhengzhou 451191, China; xxyue1115@126.com

**Keywords:** chalcone-1,2,3-triazole-azole, synthesis, antiproliferative, morphological changes

## Abstract

A series of novel chalcone-1,2,3-triazole-azole hybrids were designed, synthesized and evaluated for their antiproliferative activity against three selected cancer cell lines (SK-N-SH, EC-109 and MGC-803). Most of the synthesized compounds exhibited moderate to good activity against all the cancer cell lines selected. Particularly, compound **I-21** showed the most excellent antiproliferative activity with an IC_50_ value of 1.52 μM against SK-N-SH cancer cells. Further mechanism studies revealed that compound **I-21** induced morphological changes of SK-N-SH cancer cells possibly by inducing apoptosis. Novel chalcone-1,2,3-triazole-azole derivatives in this work might be a series of promising lead compounds to develop anticancer agents for treating neuroblastoma.

## 1. Introduction

Cancer, being one of the leading causes of death globally, causes a great burden to both the society and single human lives as a whole. Although there have been progresses in the development of treatment and prevention of cancer, the success to treat cancer remains a challenge [1]. Therefore, there is still an urgent need to search for novel anticancer agents that have broader spectrum of cytotoxicity to tumor cells [2].

**Figure 1:**
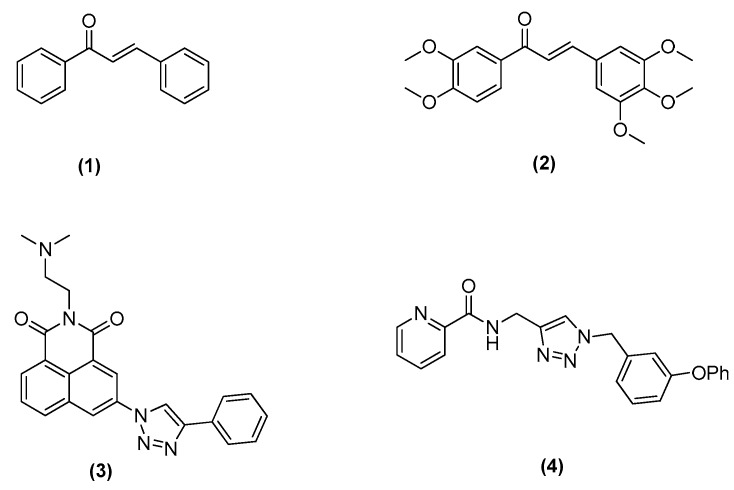
Structures of chalcone and 1,2,3-triazole derivates as antitumor agents previously reported.

Chalcones are abundant in edible plants where they are considered to be the precursors of flavonoids or isoflavonoids [3] and constitute an important group of natural and synthetic products with wide range of pharmacological activities as antibacterial [4,5], anti-tumor [6,7], anti-inflammatory [8,9], antifungal and antioxidant agents [10,11]. For example, a chalcone without substituent groups (**1**) is cytotoxic to MCF-7 cell line with an IC_50_ of 6.88 μg/mL [12]. A combretastatin-like chalcone (**2**) as the inhibitor of microtubule polymerization, exhibited the excellent cytotoxic activity against K562 cells with an IC_50_ of 1.1 μM [13] (Figure 1).

1,2,3-triazole is a considered privileged scaffold in drug discovery with a wide array of biological activities as anti-fungal [14], anti-bacterial [15], anti-allergic [16], anti-HIV [17], anti-tubercular [18] and anti-inflammatory agents [19]. Recent research of its pharmacological effects became much more appealing and promising for the design of anticancer agents. Compound (**3**), a 1,2,3-triazol-naphthalimide hybrid, showed IC_50_ values of 0.348 and 0.258 μM against MCF-7 and SMMC-7721 cell lines, respectively [20]. N-((1-Benzyl-1*H*-1,2,3-triazol-4-yl)methyl) arylamide was identified as a proprietary small molecule scaffold for potential antitumor agents by M.J. Miller group and compound (**4**) exhibited an IC_50_ of 46 nM against MCF-7 cancer cell line [21] (Figure 1).

**Figure 2:**
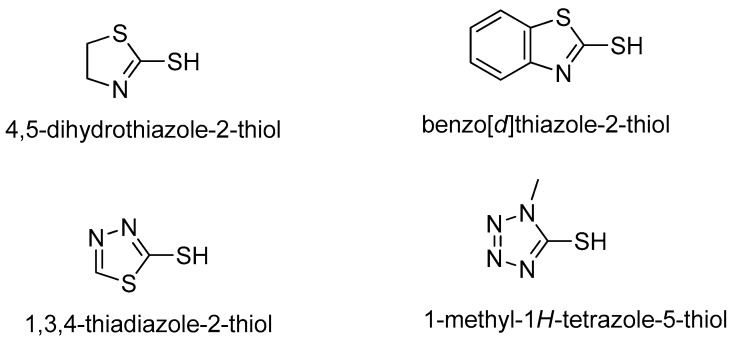
Structures of heterocyclic azoles.

On the other hand, the heterocyclic azoles, including 4,5-dihydrothiazole-2-thiol, benzo[*d*]thiazole-2-thiol, 1,3,4-thiadiazole-2-thiol, and 1-methyl-1*H*-tetrazole-5-thiol (Figure 2), represent one of the most useful cores of anticancer agents, with a wide range of activities against various cancers [22]. These above interesting findings and our continuous quest to identify more potent analogues, led to the molecular hybridization of chalcone, 1,2,3-trizole, and different azoles to integrate them in one molecular platform to generate a new hybrid architecture with the aim of exploring the impact of such modification on the anticancer agents [23,24].

**Figure 3:**
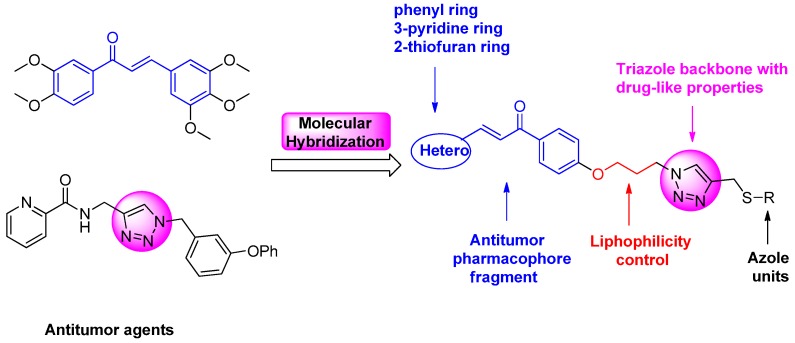
Illustration of the design strategy for target compounds.

The designed scaffold (Figure 3) has four parts: 1,2,3-trizole as a central backbone, attachment of chalcone or heterocycle chalcone to a trizole unit to enchance desired pharmacophoric behavior with druglike properties, a long-chain alkoxyl group for lipophilicity, and azole units to the other side of the triazole core. In this paper we report the synthesis and evaluation of novel chalcone-1,2,3-triazole-azoles as new class of antiproliferative agents.

In totally, a series of novel chalcone-1,2,3-triazole-azole derivatives were successfully synthesized, and their structures characterized by ^1^H-NMR, ^13^C-NMR, HRMS. Their *in vitro* antiproliferative activities were then tested, using a MTT assay, against three selected cancer cell lines (SK-N-SH, EC-109 and MGC-803) and compared with the well-known anticancer drug 5-fluorouracil. Most of the synthesized compounds exhibited moderate to good activity against all the cancer cell lines selected. Particularly, the promising compound **I-21** showed the most excellent antiproliferative activity with an IC_50_ value of 1.52 μM against SK-N-SH cancer cells.

## 2. Results and Discussion

### 2.1. Chemistry

**Scheme 1:**
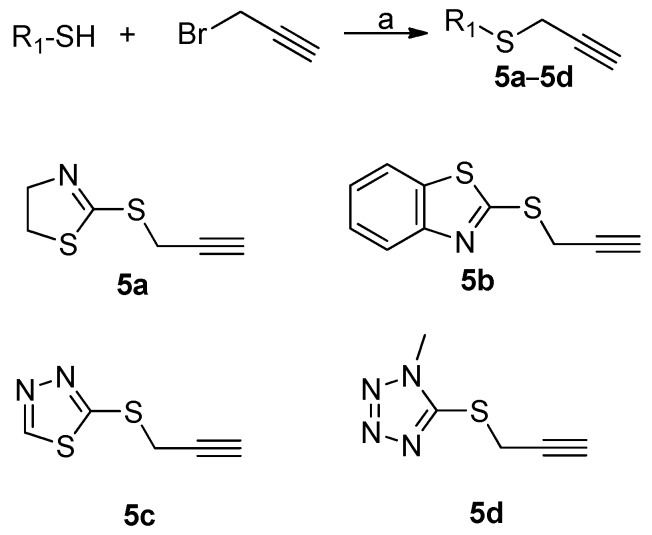
Synthesis of alkyne intermediates. *Reagents and conditions*: (**a**) K_2_CO_3_, acetone, rt.

**Scheme 2:**
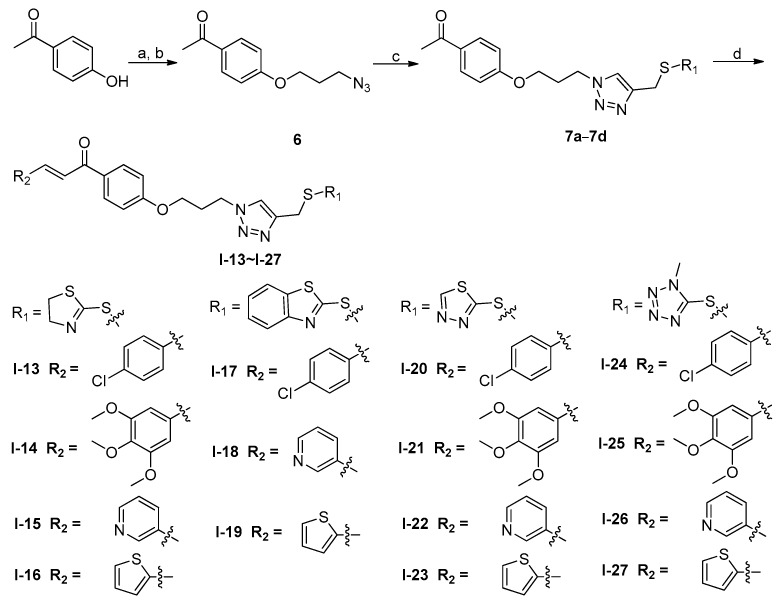
Synthesis of chalcone-1,2,3-triazole-azole analogues. *Reagents and conditions*: (**a**) K_2_CO_3_, 1,3-dibromopropane, acetone, reflux; (**b**) TBAB, NaN_3_, acetone, H_2_O, reflux; (**c**) alkyne intermediates, CuSO_4_.5H_2_O, sodium ascorbate, THF–H_2_O (1:1), rt; (**d**) substituted acetophenone, NaOH, EtOH, rt.

Alkyne intermediates **5a**–**5d** were synthesized as shown in Scheme 1. Commercially available azoles were treated with propargyl bromide to provide **5a**–**5d**. The general route for the synthesis of the target chalcone-1,2,3-triazole-azole analogues (**I**-**1**–**I**-**27**) was depicted in Scheme 2. Commercially available 1,3-dibromopropane was reacted with *p*-hydroxyacetophenone in the presence of potassium carbonate and NaN_3_ to form compound **6**, which was subjected to click reaction with appropriately alkyne intermediates to afford **7a**–**7d** with good yields. **7a**–**7d** were converted to target compounds **I**-**13**–**I**-**27** with substituted aromatic aldehyde at room tempeture in a NaOH/EtOH solution. Alkyne intermediates **5a**–**5d** and trizole intermediates **7a**–**7d** were easily obtained with the mature reaction conditions developed by our group [22]. The structures of the targeted compounds were characterized using spectral methods, and all spectral data corroborated the assumed structures.

### 2.2. Antiproliferative Activity and Structure Activity Relationship Analysis

All synthesized compounds were evaluated for their anticancer activity against three cancer cell lines, MGC-803 (human gastric cancer cell line), SK-N-SH (human neuroendocrine cancer cell line), and HepG-2 (human esophageal cancer cell line) using MTT assay method and compared with the well-known anticancer drug 5-fluorouracil. The results are summarized as Table 1. With the exception of chalcone-1,2,3-triazole-azoles **I**-**1**–**I**-**27**, all the compounds exhibit moderate potency against all three selected cancer lines. Among them, compounds **I-14** and **I-21** shows showed broad spectrum anticancer activity with IC_50_ values ranging from 3.57 to 8.52 μM and 1.52 to 10.42 μM, respectively (Table 1).

During the structure activity relationship studies, we found that the substituent on the benzene ring of chalcone has a remarkable effect on their antiproliferative activity. Regarding the activity results of the tested compounds against SK-N-SH cell line, compounds (**I-14**, **I-21**, **I-25**) with the 3,4,5-trimethoxyphenyl group on the benzene ring of chalcone showed more potent inhibitory effects (1.52–6.98 μM) than compounds (**I-13**, **I-20**, **I-24**) with a *p*-chlorine atom (Table 1).

To evaluate whether the 3,4,5-trimethoxyphenyl ring and heterocycles of chalcones have an effect on the activity, compounds with 3-pyridine ring (**I-15**, **I-18**, **I-22**, and **I-26**), 2-thiofuran ring (**I-16**, **I-19**, **I-23** and **I-27**) were synthesized and their antiproliferative activity results were shown in Table 1. Compounds bearing 3-pyridine ring (**I-18**, **I-22**, and **I-26**) showed more potent inhibitory effects than compounds bearing 2-thiofuran ring (**I-19**, **I-23** and **I-27**). Especially, compound **I-22** exhibited the most potent inhibitory activity with an IC_50_ value of 4.45 μM against MGC-803 cells. However, the opposite trend was observed that compound **I-16** bearing 2-thiofuran ring showed more potent antiproliferative acitivity than compound **I-15** bearing 3-pyridine ring against all tested cancer cell lines. The results suggested that the heterocycle ring of chalcone affected the activity obviously. Furthermore, changing the 3,4,5-trimethoxyphenyl ring (**I-14**, 3.57 μM) to 3-pyridine ring (**I-15**, 42.36 μM) and 2-thiofuran ring (**I-16**, 19.28 μM) led to a loss of antiproliferative activity against EC-109 cells. These modifications and structure activity relationship studies revealed that the 3,4,5-trimethoxyphenyl ring of chalcone played a critical role for their inhibitory activity.

To determine whether the azole rings might affect the activity, compound with 4,5-dihydrothiazole-2-thiol (**I-13**–**I-16**), benzo[*d*]thiazole-2-thiol (**I-17**–**I-19**), 1,3,4-thiadiazole-2-thiol (**I-20**–**I-23**) and 1-methyl-1*H*-tetrazole-5-thiol (**I-24**–**I-27**) were synthesized and their anticancer activity were shown in Table 1. Replacing the 1,3,4-thiadiazole-2-thiol scaffold of compound **I-20** with 4,5-dihydrothiazole-2-thiol (**I-13**) led to a loss of activity against EC-109 cancer cells. However, changing the benzo[*d*]thiazole-2-thiol (compound **I-17**) to 1-methyl-1*H*-tetrazole-5-thiol (compound **I-26**) led to an improvement of activity against SK-N-SH, indicating the significance of the azole rings in their antiproliferative activity.

**Figure 4:**
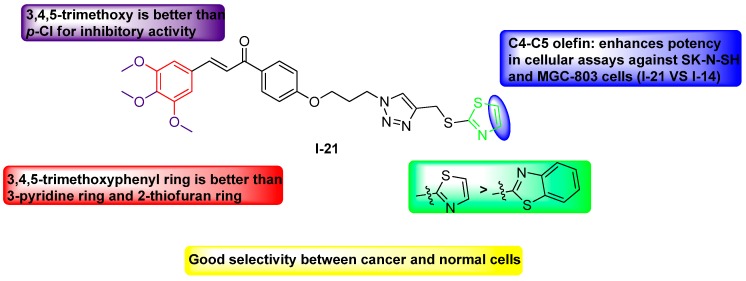
Summary of SAR of chalcone-1,2,3-triazole-azole derivatives.

Compounds **I-14** and **I-21** were further examined for possible cytotoxicity against GES-1 (normal human gastric epithelial cell line). As can be seen in Table 2, we found that compounds **I-14** and **I-21** exhibited no significant cytotoxicity against GES-1 (>64 and 43.67 μM, respectively). However, compounds **I-14** and **I-21** exhibited potent cytotoxicity against selected MGC-803 cancer cell lines (8.52 μM and 4.26 μM, respectively). The results indicated that compounds **I-14** and **I-21** had good selectivity between cancer and normal cells. The detailed illustration for structure activity relationship of target derivatives was showed in Figure 4.

### 2.3. Apoptosis Analysis with Hoechst-33258 Staning

**Figure 5:**
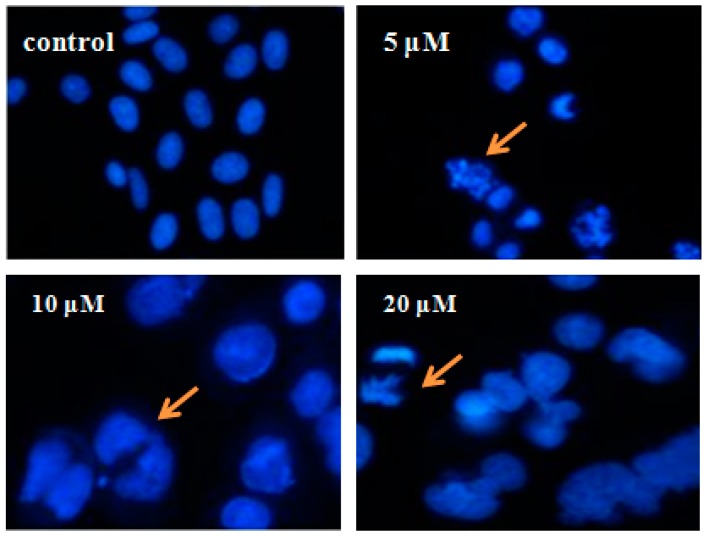
Apoptosis analysis with Hoechst-33258 staining after a 48 h treatment of **I-21** in SK-N-SH cell line at 5 μM, 10 μM and 20 μM.

Due to the most potent cytotoxic activity against three selected cancer cell lines among all synthesized derivatives [23], compound **I-21** was chosen to be further investigated regarding its mechanism of action. To explore cytotoxicity of **I-21** in SK-N-SH cells, cell apoptosis was investigated with Hoechst 33258 staining. After 24 h incubation with **I-21** at indicated concentrations, characteristic apoptotic morphological changes were on served by fluorescence microscope, including cell rounding, chromain shrinkage and formation of apoptotic bodies (Figure 5). The studies revealed that compound **I-21** induced morphological changes of SK-N-SH cancer cells possibly by inducing apoptosis.

### 2.4. Western Blots Analysis

**Figure 6:**
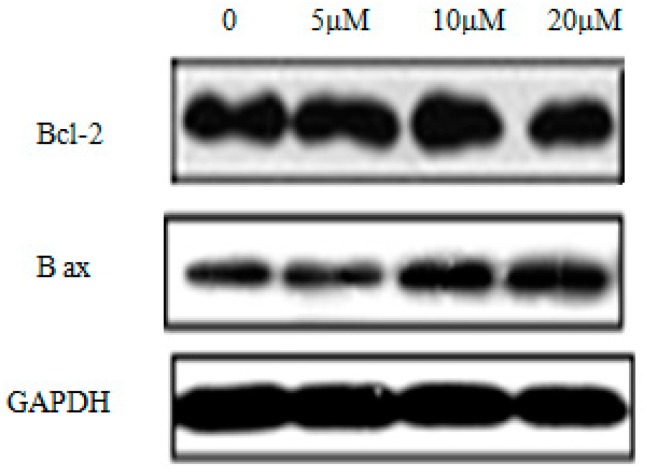
Expression of Bcl-2 and Bax in SK-N-SH cells after treatment by compound **I-21** for 48 h.

Antiproliferative agents that induce apoptosis in cancer cells are always preferred in antitumor drug discovery. Bcl-2 associated proteins have both anti-apoptotic and pro-apoptotic effects in vrious cancer cell lines. For the high potent cytotoxic activity against SK-N-SH cell line, compound **I-21** was evaluated the level of Bcl-2 (apoptotic inhibitor) and Bax (apoptotic inducer) [24]. We can see from the result that compound **I-21** increased the level of Bax and reduced the level of Bcl-2, which could promote apoptosis of SK-N-SH cells (Figure 6). Further mechanism investigations are under way and will be reported in due course.

## 3. Materials and Methods

### 3.1. General Experimental Procedures

All reagents and solvents used were of analytical grade purchased from commercial sources. Thin-layer chromatography (TLC) was carried out on glass plates coated with silica gel and visualized by UV light (254 nm) (Beijing synthware glass, Beijing, China). Melting points were determined on a Beijing Keyi XT4A apparatus and are uncorrected (Beijing, China). All NMR spectra were recorded with a Bruker DPX 400 MHz spectrometer (Agilent, Santa Clara, CA, USA), with TMS as internal standard in CDCl_3_. Chemical shifts are given as dppm values relative to TMS. For some of novel compounds, Mass spectra (MS) were recorded on Esquire 3000 mass spectrometer (Varian, Palo Alto, CA, USA) by electrospray ionization (ESI).

#### 3.1.1. General Procedure of Compounds **5a**–**5d**

To a stirred solution of mercaptan (3 mmol) in acetone (15 mL), propargyl bromide (3 mmol) and K_2_CO_3_ (3 mmol) were added carefully and the reaction mixture was refluxed for 5 h. Upon completion, the reaction mixture was concentrated under vacuum, the residue was dissolved in EtOAc (30 mL) and washed with water, brine, dried over anhydrous Na_2_SO_4_ and concentrated under vacuum to afford compounds **5a**–**5d**, which were used in the next reaction without further purification.

*2-(Prop-2-yn-1-ylthio)-4,5-dihydrothiazole* (**5a**): Colourless oil, yield: 67%. ^1^H-NMR (400 MHz, DMSO) δ 4.16 (t, *J* = 8.0 Hz, 2H), 3.94 (d, *J* = 2.6 Hz, 2H), 3.48 (t, *J* = 8.0 Hz, 2H), 3.21 (t, *J* = 2.6 Hz, 1H). ^13^C-NMR (100 MHz, DMSO) δ 161.81, 79.50, 74.13, 63.92, 35.48, 20.09. HRMS (ESI) calcd for C_6_H_8_NS_2_ [M + H]^+^: 158.0098, found: 158.0098.

*2-(Prop-2-yn-1-ylthio)benzo[d]thiazole* (**5b**): Yellow solid, yield: 87%; m.p.: 50–52 °C. ^1^H-NMR (400 MHz, DMSO) δ 7.75–7.62 (m, 2H), 7.41–7.30 (m, 2H), 4.23 (d, *J* = 2.6 Hz, 2H), 3.31 (t, *J* = 2.6 Hz, 1H). ^13^C-NMR (100 MHz, DMSO) δ 162.70, 151.38, 141.19, 124.70, 124.49, 118.46, 110.27, 79.19, 74.57, 20.33. HRMS (ESI) calcd for C_10_H_8_NS_2_ [M + H]^+^: 206.0099, found: 206.0098.

*2-(Prop-2-yn-1-ylthio)-1,3,4-thiadiazole* (**5c**): Colourless oil, yield: 80%. ^1^H-NMR (400 MHz, DMSO) δ 9.59 (dd, *J* = 3.5, 1.6 Hz, 1H), 4.23 (dd, *J* = 2.2, 1.6 Hz, 2H), 3.25 (ddd, *J* = 7.9, 4.9, 2.6 Hz, 1H). ^13^C-NMR (100 MHz, DMSO) δ 163.98, 154.52, 78.98, 74.77, 22.21. HRMS (ESI) calcd for C_5_H_5_N_2_S_2_ [M + H]^+^: 156.9897, found: 156.9894.

*1-Methyl-5-(prop-2-yn-1-ylthio)-1H-tetrazole* (**5d**): White solid, yield: 82%; m.p.: 61–62 °C. ^1^H-NMR (400 MHz, DMSO) δ 4.12 (d, *J* = 2.6 Hz, 2H), 3.99 (s, 3H), 3.26 (t, *J* = 2.6 Hz, 1H). ^13^C-NMR (100 MHz, DMSO) δ 152.29, 78.87, 74.93, 33.81, 21.58. HRMS (ESI) calcd for C_5_H_7_N_4_S [M + H]^+^: 155.0393, found: 155.0391.

#### 3.1.2. General Procedure of Compounds **7a**–**7d**

*p*-Aminoacetophenone (1 mmol), 1,3-dibromopropane (1 mmol) and K_2_CO_3_ (1 mmol) were dissolved in acetone (10 mL). The mixture was refluxed for 3 h, then sodium azide were added and the reaction mixture was refluxed for another 2 h. Upon completion, the reaction mixture was concentrated under vacuum, the residue was dissolved in EtOAc (20 mL) and washed with water, brine, dried over anhydrous MgSO_4_ and concentrated under vacuum to afford compound **6**, which were used in the next reaction without further purification. compound **6** (1.05 mmol), alkyne intermediates (1 mmol), CuSO_4_·5H_2_O (0.2 mmol) and sodium ascorbate (0.1 mmol) were dissolved in THF/H_2_O (5 mL/5 mL) to stir for 7 h at room temperature. Upon completion, the precipitated product was filtered to afford the crude product **7a–7d** without further purification.

*1-(4-(3-Azidopropoxy)phenyl)ethanone* (**6**): Colourless oil, yield: 74%. ^1^H-NMR (400 MHz, DMSO) δ 8.08–7.77 (m, 2H), 7.14–6.92 (m, 2H), 4.13 (t, *J* = 6.2 Hz, 2H), 3.53 (t, *J* = 6.7 Hz, 2H), 2.52 (s, 3H), 2.02 (p, *J* = 6.4 Hz, 2H). ^13^C-NMR (100 MHz, DMSO) δ 195.99, 162.19, 130.35, 129.99, 114.13, 64.96, 47.63, 28.01, 26.12. HRMS (ESI) calcd for C_11_H_14_N_3_O_2_ [M + H]^+^: 220.1086, found: 220.1086.

*1-(4-(3-(4-(((4,5-Dihydrothiazol-2-yl)thio)methyl)-1H-1,2,3-triazol-1-yl)propoxy)phenyl)ethanone* (**7a**): White solid, yield: 69%; m.p.: 110–112 °C. ^1^H-NMR (400 MHz, DMSO) δ 8.04 (s, 1H), 7.92 (d, *J* = 8.9 Hz, 2H), 7.00 (d, *J* = 8.9 Hz, 2H), 4.52 (t, *J* = 6.9 Hz, 2H), 4.39 (s, 2H), 4.15 (t, *J* = 8.0 Hz, 2H), 4.06 (t, *J* = 6.0 Hz, 2H), 3.44 (t, *J* = 8.0 Hz, 2H), 2.51 (s, 3H), 2.29 (p, *J* = 6.5 Hz, 2H). ^13^C-NMR (100 MHz, DMSO) δ 196.23, 162.49, 162.14, 142.65, 130.42, 130.02, 123.71, 114.25, 64.91, 63.93, 46.49, 35.30, 29.25, 26.61, 26.36. HRMS (ESI) calcd for C_17_H_21_N_4_O_2_S_2_ [M + H]^+^: 377.1107, found: 377.1106.

*1-(4-(3-(4-((Benzo[d]thiazol-2-ylthio)methyl)-1H-1,2,3-triazol-1-yl)propoxy)phenyl)ethanone* (**7b**): White solid, yield: 73%; m.p.: 121–122 °C. ^1^H-NMR (400 MHz, DMSO) δ 8.19 (s, 1H), 7.89 (d, *J* = 8.7 Hz, 2H), 7.75–7.54 (m, 2H), 7.45–7.22 (m, 2H), 6.96 (d, *J* = 8.7 Hz, 2H), 4.68 (s, 2H), 4.52 (t, *J* = 6.9 Hz, 2H), 4.04 (t, *J* = 6.0 Hz, 2H), 2.51 (s, 3H), 2.39–2.17 (m, 2H). ^13^C-NMR (100 MHz, DMSO) δ 196.19, 163.55, 162.10, 151.33, 141.24, 130.38, 129.99, 124.59, 124.31, 123.93, 118.33, 114.19, 110.21, 64.91, 46.58, 29.22, 26.51, 26.34. HRMS (ESI) calcd for C_21_H_21_N_4_O_2_S_2_ [M + H]^+^: 425.1106, found: 425.1106.

*1-(4-(3-(4-(((1,3,4-Thiadiazol-2-yl)thio)methyl)-1H-1,2,3-triazol-1-yl)propoxy)phenyl)ethanone* (**7c**): White solid, yield: 82%; m.p.: 132–135 °C. ^1^H-NMR (400 MHz, DMSO) δ 9.52 (s, 1H), 8.13 (s, 1H), 7.91 (d, *J* = 8.8 Hz, 2H), 6.99 (d, *J* = 8.8 Hz, 2H), 4.63 (s, 2H), 4.52 (t, *J* = 6.9 Hz, 2H), 4.05 (t, *J* = 6.0 Hz, 2H), 2.51 (s, 3H), 2.38–2.17 (m, 2H). ^13^C-NMR (100 MHz, DMSO) δ 196.23, 164.68, 162.12, 154.24, 142.14, 130.42, 130.02, 123.95, 114.24, 64.89, 46.56, 29.25, 28.43, 26.37. HRMS (ESI) calcd for C_16_H_18_N_5_O_2_S_2_ [M + H]^+^: 376.0902, found: 376.0902.

*1-(4-(3-(4-(((1-Methyl-1H-tetrazol-5-yl)thio)methyl)-1H-1,2,3-triazol-1-yl)propoxy)phenyl)ethanone* (**7d**): Colourless oil, yield: 63%. ^1^H-NMR (400 MHz, DMSO) δ 8.11 (s, 1H), 7.92 (d, *J* = 8.8 Hz, 2H), 7.00 (d, *J* = 8.8 Hz, 2H), 4.59 (s, 2H), 4.54 (s, 2H), 4.15–4.01 (m, 2H), 3.91 (s, 3H), 2.52 (s, 3H), 2.30 (p, *J* = 6.5 Hz, 2H). ^13^C-NMR (100 MHz, DMSO) δ 208.28, 196.16, 162.10, 152.92, 142.06, 130.38, 123.87, 114.20, 68.47, 64.84, 55.76, 46.53, 31.99, 29.53. HRMS (ESI) calcd for C_16_H_20_N_7_O_2_S [M + H]^+^: 374.1395, found: 374.1399.

#### 3.1.3. General Procedure of Compounds **I-13**–**I-27**

Compound **7a**–**7d** (1 mmol), substituted aromatic aldehyde (1 mmol) and NaOH (1 mmol) were dissolved in ethanol (10 mL) to stir at room temperature. The reaction was monitored by TLC till the reaction was finished. Upon completion, the reaction mixture was concentrated under vacuum, the residue was dissolved in EtOAc and washed with water, brine, dried over anhydrous Na_2_SO_4_ and concentrated under vacuum to afford compounds **I-13**–**I-27** which were purified with column chromatography on silica gel (hexane/EtOAc = 6/1).

*(E)-3-(4-Chlorophenyl)-1-(4-(3-(4-(((4,5-dihydrothiazol-2-yl)thio)methyl)-1H-1,2,3-triazol-1-yl)propoxy)phenyl)prop-2-en-1-one* (**I-13**): White solid, yield: 67%; m.p. 151–153 °C. ^1^H-NMR (400 MHz, CDCl_3_) δ 8.03 (d, *J* = 8.7 Hz, 2H), 7.75 (d, *J* = 15.6 Hz, 1H), 7.58 (d, *J* = 8.4 Hz, 2H), 7.54 (s, 1H), 7.51 (d, *J* = 15.7 Hz, 1H), 7.39 (d, *J* = 8.3 Hz, 2H), 6.96 (d, *J* = 8.7 Hz, 2H), 4.57 (t, *J* = 6.7 Hz, 2H), 4.41 (s, 2H), 4.14 (t, *J* = 8.0 Hz, 2H), 4.04 (t, *J* = 5.6 Hz, 2H), 3.36 (t, *J* = 8.0 Hz, 2H), 2.48–2.39 (m, 2H). ^13^C-NMR (100 MHz, CDCl_3_) δ 188.31, 164.79, 162.31, 144.36, 142.63, 136.25, 133.50, 131.29, 130.90, 129.56, 129.22, 123.21, 122.17, 114.33, 64.35, 64.10, 35.81, 29.71, 27.09. HRMS (ESI) calcd for C_24_H_23_ClN_4_O_2_S_2_ [M + H]^+^: 498.0953, found: 498.0951.

*(E)-1-(4-(3-(4-(((4,5-Dihydrothiazol-2-yl)thio)methyl)-1H-1,2,3-triazol-1-yl)propoxy)phenyl)-3-(3,4,5-trimethoxyphenyl)prop-2-en-1-one* (**I-14**): White solid, yield: 51%; m.p. 169–171 °C. ^1^H-NMR (400 MHz, CDCl_3_) δ 7.95 (d, *J* = 8.5 Hz, 2H), 7.64 (d, *J* = 15.5 Hz, 1H), 7.48 (s, 1H), 7.34 (d, *J* = 15.5 Hz, 1H), 6.88 (d, *J* = 8.5 Hz, 2H), 6.79 (s, 2H), 4.50 (t, *J* = 6.6 Hz, 2H), 4.33 (s, 2H), 4.06 (t, *J* = 7.9 Hz, 2H), 3.96 (t, *J* = 5.5 Hz, 2H), 3.85 (s, 6H), 3.82 (s, 3H), 3.28 (t, *J* = 7.9 Hz, 2H), 2.35 (dt, *J* = 11.8, 5.9 Hz, 2H). ^13^C-NMR (100 MHz, CDCl_3_) δ 188.64, 164.84, 162.17, 153.47, 144.36, 140.32, 131.42, 130.87, 130.50, 123.21, 121.11, 114.27, 105.62, 64.33, 64.08, 61.01, 56.25), 46.97, 35.79, 29.70, 27.06. HRMS (ESI) calcd for C_27_H_30_N_4_O_5_S_2_ [M + H]^+^: 554.1659, found: 554.1658.

*(E)-1-(4-(3-(4-(((4,5-Dihydrothiazol-2-yl)thio)methyl)-1H-1,2,3-triazol-1-yl)propoxy)phenyl)-3-(pyridin-3-yl)prop-2-en-1-one* (**I-15**): White solid, yield: 56%; m.p. 149–151 °C. ^1^H-NMR (400 MHz, CDCl_3_) δ 8.86 (d, *J* = 1.8 Hz, 1H), 8.62 (m, 1H), 8.04 (d, *J* = 8.8 Hz, 2H), 7.96 (d, *J* = 7.9 Hz, 1H), 7.78 (d, *J* = 15.8 Hz, 1H), 7.62 (d, *J* = 15.7 Hz, 1H), 7.56 (s, 1H), 7.37 (dd, *J* = 7.9, 4.8 Hz, 1H), 6.97 (d, *J* = 8.8 Hz, 2H), 4.58 (t, *J* = 6.7 Hz, 2H), 4.41 (s, 2H), 4.15 (t, *J* = 8.0 Hz, 2H), 4.05 (t, *J* = 5.7 Hz, 2H), 3.36 (t, *J* = 8.0 Hz, 2H), 2.48–2.40 (m, 2H). ^13^C-NMR (100 MHz, CDCl_3_) δ 187.93, 164.74, 162.45, 150.97, 149.90, 144.34, 140.24, 134.59, 130.92, 123.70, 123.19, 114.39, 64.40, 64.09, 46.95, 35.80, 29.69, 27.07. HRMS (ESI) calcd for C_23_H_23_N_5_O_2_S_2_ [M + H]^+^: 465.1293, found: 465.1293.

*(E)-1-(4-(3-(4-(((4,5-Dihydrothiazol-2-yl)thio)methyl)-1H-1,2,3-triazol-1-yl)propoxy)phenyl)-3-(thiophen-2-yl)prop-2-en-1-one* (**I-16**): White solid, yield: 61%; m.p. 104–106 °C. ^1^H-NMR (400 MHz, CDCl_3_) δ 8.01 (d, *J* = 8.8 Hz, 2H), 7.93 (d, *J* = 15.2 Hz, 1H), 7.55 (s, 1H), 7.41 (d, *J* = 5.0 Hz, 1H), 7.33 (d, *J* = 15.5 Hz, 2H), 7.12–7.06 (m, 1H), 6.95 (d, *J* = 8.8 Hz, 2H), 4.57 (t, *J* = 6.7 Hz, 2H), 4.41 (s, 2H), 4.13 (t, *J* = 8.0 Hz, 2H), 4.03 (t, *J* = 5.7 Hz, 2H), 3.35 (t, *J* = 8.0 Hz, 2H), 2.48–2.38 (m, 2H). ^13^C-NMR (100 MHz, CDCl_3_) δ 188.01, 164.79, 162.19, 144.38, 140.49, 136.61, 131.90, 131.39, 130.78, 128.62, 128.36, 123.23, 120.50, 114.28, 64.31, 64.09, 46.97, 35.81, 29.66, 27.09. HRMS (ESI) calcd for C_22_H_22_N_4_O_2_S_3_ [M + H]^+^: 470.0906, found: 470.0905.

*(E)-1-(4-(3-(4-((Benzo[d]thiazol-2-ylthio)methyl)-1H-1,2,3-triazol-1-yl)propoxy)phenyl)-3-(4-phenyl)prop-2-en-1-one* (**I-17**): White solid, yield: 59%; m.p. 172–174 °C. ^1^H-NMR (400 MHz, CDCl_3_) δ 7.99 (d, *J* = 8.7 Hz, 2H), 7.84 (d, *J* = 8.1 Hz, 1H), 7.80–7.72 (m, 2H), 7.66 (s, 1H), 7.59 (d, *J* = 8.4 Hz, 2H), 7.51 (d, *J* = 15.6 Hz, 1H), 7.42 (m, 3H), 7.32 (d, *J* = 7.5 Hz, 1H), 6.90 (d, *J* = 8.8 Hz, 2H), 4.70 (s, 2H), 4.57 (t, *J* = 6.7 Hz, 2H), 4.01 (t, *J* = 5.7 Hz, 2H), 2.47–2.38 (m, 2H). ^13^C-NMR (100 MHz, CDCl_3_) δ 188.30, 165.84, 162.24, 152.96, 142.60, 136.26, 133.53, 131.33, 130.78, 129.55, 129.24, 128.48, 126.13, 124.44, 122.20, 121.43, 121.16, 114.23, 64.27, 47.00, 29.67, 27.71. HRMS (ESI) calcd for C_28_H_23_ClN_4_O_2_S_2_ [M + H]^+^: 546.0951, found: 546.0951.

*(E)-1-(4-(3-(4-((Benzo[d]thiazol-2-ylthio)methyl)-1H-1,2,3-triazol-1-yl)propoxy)phenyl)-3-(pyridin-3-yl)prop-2-en-1-one* (**I-18**): White solid, yield: 69%; m.p. 144–146 °C. ^1^H-NMR (400 MHz, CDCl_3_) δ 8.86 (s, 1H), 8.62 (d, *J* = 2.5 Hz, 1H), 7.96 (m, 3H), 7.82 (d, *J* = 8.1 Hz, 1H), 7.77 (d, *J* = 15.8 Hz, 1H), 7.72 (d, *J* = 7.9 Hz, 1H), 7.66 (s, 1H), 7.59 (d, *J* = 15.7 Hz, 1H), 7.38 (m, 2H), 7.29 (d, *J* = 7.3 Hz, 1H), 6.89 (d, *J* = 8.6 Hz, 2H), 4.67 (s, 2H), 4.55 (t, *J* = 6.6 Hz, 2H), 4.00 (t, *J* = 5.6 Hz, 2H), 2.46–2.35 (m, 2H). ^13^C-NMR (100 MHz, CDCl_3_) δ 187.86, 165.79, 162.38, 152.93, 150.93, 149.87, 144.02, 140.14, 135.41, 134.56, 130.91, 126.11, 124.43, 123.72, 123.42, 121.4, 121.15, 114.30, 64.35, 46.99, 29.65, 27.73. HRMS (ESI) calcd for C_27_H_23_N_5_O_2_S_2_ [M + H]^+^: 513.1293, found: 513.1293.

*(E)-1-(4-(3-(4-((Benzo[d]thiazol-2-ylthio)methyl)-1H-1,2,3-triazol-1-yl)propoxy)phenyl)-3-(thiophen-2-yl)prop-2-en-1-one* (**I-19**): White solid, yield: 55%; m.p. 144–147 °C. ^1^H-NMR (400 MHz, CDCl_3_) δ 7.99–7.90 (m, 3H), 7.82 (d, *J* = 8.1 Hz, 1H), 7.72 (d, *J* = 7.9 Hz, 1H), 7.64 (s, 1H), 7.45–7.28 (m, 5H), 7.12–7.06 (m, 1H), 6.87 (d, *J* = 8.8 Hz, 2H), 4.67 (s, 2H), 4.55 (t, *J* = 6.7 Hz, 2H), 3.98 (t, *J* = 5.7 Hz, 2H), 2.45–2.35 (m, 2H). ^13^C-NMR (100 MHz, CDCl_3_) δ 187.95, 165.83, 162.12, 152.96, 144.11, 140.53, 136.52, 135.46, 131.86, 131.34, 130.72, 128.94, 128.47, 126.13, 124.44, 123.45, 121.44, 121.16, 120.56, 114.18, 64.26, 47.00, 29.67, 27.74. HRMS (ESI) calcd for C_26_H_22_N_4_O_2_S_3_ [M + H]^+^: 518.0905, found: 518.0905.

*(E)-1-(4-(3-(4-(((1,3,4-Thiadiazol-2-yl)thio)methyl)-1H-1,2,3-triazol-1-yl)propoxy)phenyl)-3-(4-chlorophenyl)prop-2-en-1-one* (**I-20**): White solid, yield: 68%; m.p. 143–145 °C. ^1^H-NMR (400 MHz, CDCl_3_) δ 9.00 (s, 1H), 8.01 (d, *J* = 8.7 Hz, 2H), 7.74 (t, *J* = 7.8 Hz, 2H), 7.57 (d, *J* = 8.4 Hz, 2H), 7.51 (d, *J* = 15.6 Hz, 1H), 7.38 (d, *J* = 8.4 Hz, 2H), 6.93 (d, *J* = 8.7 Hz, 2H), 4.67 (s, 2H), 4.56 (t, *J* = 6.8 Hz, 2H), 4.02 (t, *J* = 5.7 Hz, 2H), 2.47–2.37 (m, 2H). HRMS (ESI) calcd for C_23_H_20_ClN_5_O_2_S_2_ [M + H]^+^: 497.0747, found: 497.0747.

*(E)-1-(4-(3-(4-(((1,3,4-Thiadiazol-2-yl)thio)methyl)-1H-1,2,3-triazol-1-yl)propoxy)phenyl)-3-(3,4,5-trimethoxyphenyl)prop-2-en-1-one* (**I-21**): White solid, yield: 56%; m.p. 124–127 °C. ^1^H-NMR (400 MHz, CDCl_3_) δ 9.00 (s, 1H), 8.02 (d, *J* = 8.7 Hz, 2H), 7.72 (d, *J* = 15.8 Hz, 2H), 7.43 (d, *J* = 15.6 Hz, 1H), 6.93 (d, *J* = 8.6 Hz, 2H), 6.88 (s, 2H), 4.67 (s, 2H), 4.56 (t, *J* = 6.7 Hz, 2H), 4.02 (t, *J* = 5.7 Hz, 2H), 3.93 (s, 6H), 3.90 (s, 3H), 2.47–2.37 (m, 2H). ^13^C-NMR (100 MHz, CDCl_3_) δ 188.67, 165.02, 162.13, 153.47, 151.88, 144.33, 143.35, 140.31, 131.49, 130.85, 130.51, 123.81, 121.17, 114.26, 105.63, 64.35. HRMS (ESI) calcd for C_26_H_27_N_5_O_5_S_2_ [M + H]^+^: 553.1454, found: 553.1454.

*(E)-1-(4-(3-(4-(((1,3,4-Thiadiazol-2-yl)thio)methyl)-1H-1,2,3-triazol-1-yl)propoxy)phenyl)-3-(pyridin-3-yl)prop-2-en-1-one* (**I-22**): White solid, yield: 58%; m.p. 146–149 °C. ^1^H-NMR (400 MHz, CDCl_3_) δ 9.00 (s, 1H), 8.86 (s, 1H), 8.63 (d, *J* = 3.9 Hz, 1H), 8.03 (d, *J* = 8.8 Hz, 2H), 7.96 (d, *J* = 7.9 Hz, 1H), 7.78 (d, *J* = 15.7 Hz, 1H), 7.74 (s, 1H), 7.61 (d, *J* = 15.8 Hz, 1H), 7.37 (dd, *J* = 7.8, 4.8 Hz, 1H), 6.95 (d, *J* = 8.8 Hz, 2H), 4.67 (s, 2H), 4.57 (t, *J* = 6.8 Hz, 2H), 4.04 (t, *J* = 5.7 Hz, 2H), 2.47–2.38 (m, 2H). ^13^C-NMR (100 MHz, CDCl_3_) δ 188.01, 165.02, 162.42, 151.85, 150.95, 149.89, 143.36, 140.25, 134.61, 131.39, 131.00, 123.74, 114.39, 64.37, 47.05, 29.68, 28.16. HRMS (ESI) calcd for C_22_H_20_N_6_O_2_S_2_ [M + H]^+^: 464.1089, found: 464.1089.

*(E)-1-(4-(3-(4-(((1,3,4-Thiadiazol-2-yl)thio)methyl)-1H-1,2,3-triazol-1-yl)propoxy)phenyl)-3-(thiophen-2-yl)prop-2-en-1-one* (**I-23**): White solid, yield: 72%; m.p. 124–126 °C.^1^H-NMR (400 MHz, CDCl_3_) δ 8.99 (s, 1H), 7.99 (d, *J* = 8.8 Hz, 2H), 7.93 (d, *J* = 15.3 Hz, 1H), 7.72 (s, 1H), 7.41 (d, *J* = 5.0 Hz, 1H), 7.33 (d, *J* = 15.1 Hz, 2H), 7.11–7.07 (m, 1H), 6.92 (d, *J* = 8.8 Hz, 2H), 4.67 (s, 2H), 4.56 (t, *J* = 6.8 Hz, 2H), 4.01 (t, *J* = 5.7 Hz, 2H), 2.46–2.37 (m, 2H).^13^C-NMR (100 MHz, CDCl_3_) δ 188.06, 162.16, 151.86, 143.33, 140.51, 136.60, 131.87, 131.38, 130.76, 128.59, 128.35, 123.83, 120.56 114.27, 64.30, 47.06, 29.69, 28.18. HRMS (ESI) calcd for C_21_H_19_N_5_O_2_S_3_ [M + H]^+^: 469.0703, found: 469.0701.

*(E)-3-(4-Chlorophenyl)-1-(4-(3-(4-(((1-methyl-1H-tetrazol-5-yl)thio)methyl)-1H-1,2,3-triazol-1-yl)propoxy)phenyl)prop-2-en-1-one* (**I-24**): White solid, yield: 81%; m.p. 149–150 °C. ^1^H-NMR (400 MHz, CDCl_3_) δ 8.02 (d, *J* = 8.8 Hz, 2H), 7.78 (s, 1H), 7.74 (d, *J* = 15.7 Hz, 1H), 7.58 (d, *J* = 8.5 Hz, 2H), 7.52 (d, *J* = 15.6 Hz, 1H), 7.39 (d, *J* = 8.4 Hz, 2H), 6.94 (d, *J* = 8.8 Hz, 2H), 4.61 (s, 2H), 4.56 (t, *J* = 6.8 Hz, 2H), 4.03 (t, *J* = 5.7 Hz, 2H), 3.86 (s, 3H), 2.41 (p, *J* = 6.4 Hz, 2H). ^13^C-NMR (101 MHz, CDCl_3_) δ 188.37, 162.25, 153.71, 142.69, 136.23, 133.52, 131.29, 130.88, 129.57), 129.21, 124.00, 122.22, 114.31, 64.30, 47.09, 33.45, 29.69, 27.42. HRMS (ESI) calcd for C_23_H_22_ClN_7_O_2_S [M + H]^+^: 495.1245, found: 495.1244.

*(E)-1-(4-(3-(4-(((1-Methyl-1H-tetrazol-5-yl)thio)methyl)-1H-1,2,3-triazol-1-yl)propoxy)phenyl)-3-(3,4,5-trimethoxyphenyl)prop-2-en-1-one* (**I-25**): White solid, yield: 45%; m.p. 105–107 °C. ^1^H-NMR (400 MHz, CDCl_3_) δ 8.02 (d, *J* = 8.8 Hz, 2H), 7.78 (s, 1H), 7.72 (d, *J* = 15.6 Hz, 1H), 7.43 (d, *J* = 15.6 Hz, 1H), 6.94 (d, *J* = 8.8 Hz, 2H), 6.88 (s, 2H), 4.61 (s, 2H), 4.56 (t, *J* = 6.8 Hz, 2H), 4.03 (t, *J* = 5.7 Hz, 2H), 3.93 (s, 6H), 3.90 (s, 3H), 3.86 (s, 3H), 2.46–2.36 (m, 2H). ^13^C-NMR (100 MHz, CDCl_3_) δ 188.66, 162.12, 153.70, 153.45, 144.31, 142.75, 140.25, 131.47, 130.84, 130.52, 123.99, 121.16, 114.26, 105.60, 64.28, 61.00, 56.24, 47.09, 33.44, 29.67, 27.41. HRMS (ESI) calcd for C_26_H_29_N_7_O_5_S [M + H]^+^: 551.1953, found: 551.1951.

*(E)-1-(4-(3-(4-(((1-Methyl-1H-tetrazol-5-yl)thio)methyl)-1H-1,2,3-triazol-1-yl)propoxy)phenyl)-3-(pyridin-3-yl)prop-2-en-1-one* (**I-26**)**:** White solid, yield: 75%; m.p. 151–153 °C. ^1^H-NMR (400 MHz, CDCl_3_) δ 8.88 (s, 1H), 8.64 (s, 1H), 8.03 (d, *J* = 8.8 Hz, 2H), 7.97 (d, *J* = 7.9 Hz, 1H), 7.78 (t, *J* = 7.9 Hz, 2H), 7.62 (d, *J* = 15.7 Hz, 1H), 7.38 (dd, *J* = 7.1, 4.8 Hz, 1H), 6.96 (d, *J* = 8.8 Hz, 2H), 4.62 (s, 2H), 4.56 (t, *J* = 6.8 Hz, 2H), 4.04 (t, *J* = 5.7 Hz, 2H), 3.86 (s, 3H), 2.47–2.37 (m, 2H). ^13^C-NMR (100MHz, CDCl_3_) δ 188.01, 162.39, 153.71, 150.96, 149.93, 142.77, 140.25, 134.58, 131.00, 123.99, 123.67, 114.39, 64.32, 47.08, 33.45, 29.68, 27.41. HRMS (ESI) calcd for C_22_H_22_N_8_O_2_S [M + H]^+^: 462.1587, found: 462.1586.

*(E)-1-(4-(3-(4-(((1-Methyl-1H-tetrazol-5-yl)thio)methyl)-1H-1,2,3-triazol-1-yl)propoxy)phenyl)-3-(thiophen-2-yl)prop-2-en-1-one* (**I-27**): White solid, yield: 65%; m.p. 129–130 °C. ^1^H-NMR (400 MHz, CDCl_3_) δ 8.00 (d, *J* = 8.8 Hz, 2H), 7.93 (d, *J* = 15.3 Hz, 1H), 7.78 (s, 1H), 7.41 (d, *J* = 5.0 Hz, 1H), 7.34 (d, *J* = 15.4 Hz, 2H), 7.09 (dd, *J* = 5.0, 3.7 Hz, 1H), 6.93 (d, *J* = 8.8 Hz, 2H), 4.61 (s, 2H), 4.55 (t, *J* = 6.9 Hz, 2H), 4.03 (t, *J* = 5.7 Hz, 2H), 3.85 (s, 3H), 2.45–2.36 (m, 2H). ^13^C-NMR (100 MHz, CDCl_3_) δ 188.02, 162.14, 153.70, 142.71, 140.49, 136.56, 131.89, 131.34, 130.74, 128.61, 128.35, 123.97, 120.53, 114.27, 64.30, 47.12, 33.45, 29.69, 27.46. HRMS (ESI) calcd for C_21_H_21_N_7_O_2_S_2_ [M + H]^+^: 467.1198, found: 467.1198.

## 4. Conclusions

In summary, a series of novel chalcone-1,2,3-triazole-azole hybrids were designed, synthesized and evaluated for their anticancer activity against three selected cancer cell lines (SK-N-SH, EC-109 and MGC-803). Most of the synthesized compounds exhibited moderate to good activity against all the cancer cell lines selected. Particularly, compound **I-21** showed the most excellent anticancer activity with an IC_50_ value of 1.52 μM against SK-N-SH cancer cells. Efforts to optimize the structure of compound **I-21** to further improve its potency are ongoing.

## Figures and Tables

**Table 1:** Inhibitory results of preliminary evaluation against three cancer cell lines for the target compounds.

Comp.	IC_50_ (μM) ^a^		
	EC-109	SK-N-SH	MGC-803
**I-13**	>100	19.34 ± 0.47	37.19 ± 0.85
**I-14**	3.57 ± 0.22	5.87 ± 1.05	8.52 ± 0.56
**I-15**	42.36 ± 0.18	47.31 ± 1.57	63.84 ± 1.17
**I-16**	19.28 ± 0.79	11.19 ± 0.26	8.69 ± 0.42
**I-17**	9.54 ± 0.05	>100	38.17 ± 0.58
**I-18**	24.61 ± 3.80	14.81 ± 0.72	6.24 ± 0.05
**I-19**	38.78 ± 1.05	37.83 ± 0.91	24.35 ± 0.71
**I-20**	5.81 ± 0.21	3.75 ± 1.21	46.28 ± 1.64
**I-21**	10.42 ± 0.24	1.52 ± 0.30	4.26 ± 0.32
**I-22**	14.62 ± 1.21	12.43 ± 0.44	4.45 ± 0.82
**I-23**	57.25 ± 1.63	20.96 ± 1.81	15.09 ± 0.85
**I-24**	24.94 ± 1.09	28.37 ± 1.59	50.12 ± 0.97
**I-25**	5.28 ± 0.56	6.98 ± 0.40	5.66 ± 0.55
**I-26**	11.85 ± 0.74	4.49 ± 0.45	5.56 ± 0.45
**I-27**	>100	29.30 ± 0.85	38.12 ± 0.91
**5-FU**	10.30 ± 0.32	9.85 ± 0.76	7.14 ± 0.28

^a^ Inhibitory activity was assayed by exposure for 72 h to substances and expressed as concentration required to inhibit tumor cell proliferation by 50% (IC_50_). Data are presented as the means ± SDs of three independent experiments.

**Table 2:** Inhibitory results of chalcone-1,2,3-triazole-azoles against GES-1 cell lines. 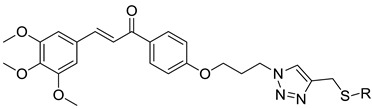

Comp.	R	IC_50_ (μM) ^a^
**I-14**	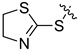	>64
**I-21**	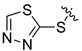	43.67 ± 1.35

^a^ Inhibitory activity was assayed by exposure for 72 h to substances and expressed as concentration required to GES-1 cell proliferation by 50% (IC_50_). Data are presented as the means ± SDs of three independent experiments.
